# Protective mechanisms of quercetin in neonatal rat brain injury induced by hypoxic‐ischemic brain damage (HIBD)

**DOI:** 10.1002/fsn3.3684

**Published:** 2023-10-13

**Authors:** Yan‐hong Xu, Jin‐bo Xu, Lu‐lu Chen, Wei Su, Qing Zhu, Guang‐lei Tong

**Affiliations:** ^1^ Anhui Provincial Children's Hospital Hefei China

**Keywords:** autophagy, HIBD, neonatal rat, NLRX1, quercetin

## Abstract

Neonatal hypoxic‐ischemic brain damage (HIBD) is a leading cause of infant mortality worldwide. This study explored whether quercetin (Que) exerts neuroprotective effects in a rat model of HIBD. A total of 36 seven‐day‐old Sprague–Dawley rats were divided into control, Que, HI, and HI + Que groups. The Rice method was used to establish HIBD in HI and HI + Que rats, which were treated with hypoxia (oxygen concentration of 8%) for 2 h after ligation of the left common carotid artery. The rats in the HI + Que group were intraperitoneally injected with Que (30 mg/kg) 1 h before hypoxia, and the rats in the Que group were only injected with the same amount of Que. Brain tissues were harvested 24 h postoperation and assessed by hematoxylin and eosin staining, 2,3,5‐triphenyltetrazolium chloride staining, and terminal deoxynucleotidyl transferase dUTP nick‐end labeling assay; relative gene and protein levels were evaluated by RT‐qPCR, IHC, or western blot (WB) assay. Brain tissue morphologies were characterized by transmission electron microscopy (TEM); LC3B protein levels were assessed by immunofluorescence staining. Escape latencies and platform crossing times were significantly improved (*p* < .05) in HI + Que groups; infarct volume significantly decreased (*p* < .001), whereas the numbers of autophagic bodies and apoptotic cells increased and decreased, respectively. Meanwhile, NLRX1, ATG7, and Beclin1 expressions were significantly upregulated, and mTOR and TIM23 expressions, LC3B protein level, and LC 3II/LC 3I ratio were significantly downregulated. Que exerted neuroprotective effects in a rat model of HIBD by regulating NLRX1 and autophagy.

## Graphical abstract

Que had improved HIBD‐induced brain injury via regulation NLRX1 to autophagy in neonatal rats.

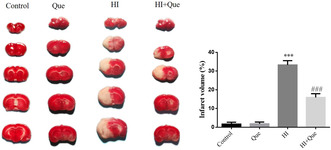

## 1 INTRODUCTION

Neonatal hypoxic‐ischemic brain damage (HIBD), also referred to as neonatal hypoxic‐ischemic encephalopathy (HIE), is a leading cause of infant mortality and irreversible long‐term neurodevelopmental impairments (Schreglmann et al., 2020). HIE‐related complications account for approximately 23% of infant deaths worldwide, impacting 700,000–1,200,000 newborns annually (Lawn et al., 2005; Narayanamurthy et al., 2021).

Understanding the pathogenesis of HIBD and its relationship to prognosis is a prerequisite for the development of effective prevention and treatment plans (Lenart, 2017). Increasing evidence suggests that mitochondrial dysfunction plays a central role in various neurological disorders (Zhou et al., 2018) as well as immature brain injury (Hagberg et al., 2014). Hypoxia impairs ATP synthesis and damages mitochondria, causing electrons in the oxidative respiratory chain to leak and generate many reactive oxygen species (ROS) and apoptotic factors that are released into neuronal cytoplasm, activating cell death pathways (Sun et al., 2015). Following HIBD induces changes in mitochondrial permeability, mitochondrial dynamics, mitophagy, and biogenesis (Demarest, Schuh, et al., 2016; Lemasters, 2005; Thornton et al., 2018). As such, mitophagy is a potential therapeutic target for the treatment of HIBD.

Nucleotide‐binding oligomerization domain, leucine‐rich repeat‐containing X1 (NLRX1) is a member of the NOD‐like receptor (NLR) family, a group of pattern recognition receptors associated with innate immunity (Nagai‐Singer et al., 2019). As the only NLR member that constitutively localizes in the mitochondria, NLRX1 is unique in several ways. The N terminus of NLRX1 lacks an effector domain and is instead replaced by a mitochondrial targeting sequence (Killackey et al., 2019), suggesting that NLRX1 coordinates mitochondrial function (Pickering & Booty, 2021). The distribution of NLRX1 in different subcellular and submitochondrial components may vary across environments and cell types (Moore et al., 2008). NLRX1 is associated with central nervous system diseases (Gharagozloo et al., 2019; Patergnani et al., 2017), ischemia–reperfusion injury (Peng et al., 2020; Zhang et al., 2020), autoimmunity (Koo et al., 2020), and cancer (Singh et al., 2019). NLRX1 can induce autophagy (Lei et al., 2012) and regulate cell death (Killackey et al., 2019; Stokman et al., 2017), especially during ischemia–reperfusion injury. However, whether NLRX1 plays a role in the injured developing brain remains unclear.

Quercetin (Que) is a natural flavonoid compound with a broad range of pharmacological activities; it may protect against oxidative stress‐induced brain injury by inhibiting cell apoptosis (Vilella et al., 2022). Yet, the effects of Que on mitochondrial function following HIE remain unknown. This study uses a rat model to explore how Que regulates mitophagy in HIBD as well as its interactions with mitochondria‐localized protein NLRX1. This investigation aims to reveal the mechanisms behind HIBD and explore the neuroprotective potential of Que.

## 2 MATERIALS AND METHODS

### 2.1 Establishment of a HIBD rat model

A total of 36 healthy 7‐day‐old Sprague–Dawley neonatal rats were purchased without sex preferences from the Animal Research Centre of Anhui Medical University; their body masses ranged from 12 to 16 g, and they were freely fed by their mothers under alternating 12‐h light/dark conditions. Each of the following four groups included nine rats: control, Que (30 mg/kg), HI, and HI + Que (30 mg/kg). The HIBD model was established using the Rice method (Rice et al., 1981) as follows: Rats were anesthetized with ether and fixed in a supine position on the operating table, their skin was disinfected with 75% alcohol, and a midline incision was made in the middle of the neck. The right common carotid artery was isolated and ligated with a silk thread; the blood vessel was severed to induce ischemic injury. The incision was sutured and disinfected again. After the ischemic surgery, the rats returned to their nest for 2 h of recovery before being placed in a 30 cm × 40 cm × 50 cm hypoxia chamber filled with a mixture of oxygen and nitrogen (8% O_2_ + 92% N_2_) at a rate of 2 L/min for 2 h to induce hypoxic injury. During hypoxia, the chamber was placed in a 37°C water bath. The rats were then returned to their original cage for rearing. Only the common carotid artery was isolated in the control and Que groups without ligation and hypoxia treatment. The Que group received an intraperitoneal injection of 30 mg/kg Que, whereas an intraperitoneal injection of 30 mg/kg Que was immediately administered in the HI + Que group once daily for seven consecutive days. An equivalent amount of physiological saline was intraperitoneally administered in the control and HI groups at the same time points. After surgery, the rats returned to their original cages and were reared by their mothers. The long‐term spatial learning and memory abilities of the 28‐day‐old rats were assessed using the Morris water maze test, after which the rats were decapitated, and brain tissues were collected.

### 2.2 Methods

#### 2.2.1 Morris water maze test

An appropriate amount of ink was added to the Morris water maze pool to render it opaque, and the water temperature was maintained at 22°C (Liu et al., 2016). Facing the wall of the pool, each rat was placed in the water maze. The time needed for the rat to enter the water, find a hidden underwater platform, and stand on it to escape was recorded daily for 5 days. On the sixth day, the platform was removed; the time needed for the rat to reach the original platform position for the first time, as well as the number of times it pierced within 120 s, were recorded.

#### 2.2.2 TTC staining

The intact brain tissues were immediately frozen in a −20°C freezer for 5 min. The frozen tissues were then coronally sectioned at 2‐mm intervals and immersed in a 1% 2,3,5‐triphenyltetrazolium chloride (TTC) solution for 10 min at 37°C in the dark. The control tissues appeared red, whereas the ischemic areas were white. Residual fluid around the brain slices was absorbed with filter paper, and digital images were captured. Image Pro Plus 6.0 software was used to quantify the infarcted area and its total volume. The percentage of infarcted volume to the total tissue volume was calculated.

#### 2.2.3 Hematoxylin and eosin staining

Formalin‐fixed brain specimens were dehydrated in a series of diluted ethanol solutions for 10 min each, cleared with xylene, and embedded in paraffin. Coronal sections were trimmed to a thickness of 5 μm and mounted on slides, which were heated to adhere to the tissue. Hematoxylin and eosin (H&E) staining was performed for 3 min, followed by dehydration with alcohol. The sections were cleared with xylene and mounted with neutral resin. Pathological changes were observed under a light microscope and assessed based on previous literature (Chen et al., 2019).

#### 2.2.4 Transmission electron microscopy characterization of neonatal rat mitochondria

Brain tissue samples were collected from each group of neonatal rats and fixed with 3% glutaraldehyde and 1.5% paraformaldehyde overnight at 4°C. After rinsing with PBS, samples were postfixed with 1% osmic acid at 4°C for 1 h. Samples were dehydrated with a graded series of ethanol (50%, 70%, 90%, and 100%) and 100% acetone; they were then infiltrated and embedded with EPON 618 resin. Ultra‐thin sections were prepared and stained with lead and uranium before they were observed and photographed using an FEI Tecnai G2 transmission electron microscope.

#### 2.2.5 Transferase dUTP nick‐end labeling (TUNEL) staining

Brain tissue samples were fixed overnight in 4% paraformaldehyde solution and embedded in paraffin; a terminal deoxynucleotidyl TUNEL assay was performed on frozen sections to detect apoptotic cells according to the manufacturer's instructions. A fluorescence microscope was used to image TUNEL‐positive cells at excitation and emission wavelengths of 405 and 525 nm, respectively. The apoptotic cell rate was calculated in six nonoverlapping high‐power fields (×200) that were randomly selected.

#### 2.2.6 RT‐qPCR assay

Total RNA was extracted from each group of cells using an RNAiso Plus kit (Takara Bio) per the manufacturer's protocol. The integrity of the extracted RNA was assessed using agarose gel electrophoresis, and the concentration and purity of RNA were determined using a nucleic acid analyzer. Then, 2 μg of total RNA was reverse transcribed, and the components were added for detection according to the instructions of the real‐time PCR reagent kit. The primer sequences are shown in Table 1. Using *GAPDH* as the internal reference, the relative expression levels of the target gene mRNA were calculated using the $2^{-\Delta \Delta C_{t}}$ method.

**TABLE 1:** The primer sequences.

Gene name	F: (5′‐3′)	R: (5′‐3′)
NLRX1	TGAAGTCCAACGCAACCTCAACAG	TCGCTCTCCACCCGCAGAAG
Beclin1	TAGCCGACCGGGAAGTA	CGACGCTCTTCACCTCA
mTOR	GGCTTCTGAAGATGCTGTCC	GAGTTCGAAGGGCAAGAGTG
ATG7	GCACTGTGAGTCGTCCAGGATTG	GTCACTGCTGCTGGCGATGG
TIM23	CTGACTGGTATGAACCCCCT	CTAGTTCAAATCTGCCTCGG
GAPDH	AGCCTCAAGATCATCAGCAATG	TGTGGTCATGAGTCCTTCCACG

#### 2.2.7 Immunohistochemical staining (IHC)

Paraffin‐embedded brain tissues were sectioned to a thickness of 5 μm, followed by dewaxing and dehydration. After three 5‐min washes with 0.01 mol PBS, samples underwent antigen retrieval using sodium citrate antigen retrieval buffer and microwave heating. They were then incubated at room temperature in the dark with 0.3% H_2_O_2_ for 10 min, followed by three 5‐min PBS washes. Samples were incubated with primary antibodies in a humidified chamber overnight at 4°C. The sections were washed with PBS and incubated at room temperature for 30 min with goat anti‐rabbit IgG antibody conjugated to horseradish peroxidase (goat anti‐rabbit IgG‐HRP, 1:1000). After additional PBS washes, the sections were stained with DAB and were observed under a microscope. The reaction was stopped with double‐distilled water at the appropriate time, followed by dehydration, clearing, and mounting with neutral resin for microscopic examination. Yellow or brownish–yellow cells were considered immunopositive. Integrated optical density (IOD) values were measured using Image Pro Plus 6.0 software.

#### 2.2.8 WB assay

A bicinchoninic acid assay was performed to quantify proteins, followed by 10% sodium dodecyl sulfate–polyacrylamide gel electrophoresis. The gel was transferred to a nitrocellulose (NC) membrane, which was blocked with 10% nonfat milk for 1 h, rinsed three times with Tris‐buffered saline with Tween 20 (TBST) for 10 min, and incubated with primary antibodies (NLRX1, 1:1000; mTOR, 1:500; Beclin1, 1:1000; ATG7, 1:1000; TIM23, 1:1000; LC3, 1:1000) overnight at 4°C in a humidified chamber. The membrane was rinsed with TBST again and incubated at room temperature for 1 h with secondary antibodies (goat anti‐rabbit IgG, 1:3000). After additional TBST washes, the membrane was incubated with enhanced chemiluminescence reagent for 5 min, wrapped in plastic film, and exposed to X‐ray film in a dark room for imaging. IOD values were calculated using ImageJ.

#### 2.2.9 Immunofluorescence staining

Mouse brain tissues were fixed in 4% paraformaldehyde for 48 h and embedded in 2.5% agarose before sectioning. The brain sections were permeabilized with 0.5% Triton X‐100 and then incubated in fetal bovine serum for 1 h. Sections were incubated with the primary antibody (LC3B, 1:500) at 4°C overnight, followed by incubation with the secondary antibody at room temperature for 2 h. Finally, samples were stained with DAPI in the dark at room temperature for 5 min. Images were captured using a fluorescence microscope and analyzed using Image Pro Plus 6.0 software.

### 2.3 Statistical analysis

All data were analyzed and plotted using SPSS 22.0 software and GraphPad Prism 8.0. Quantitative data were expressed as mean ± standard deviation (mean ± SD). Student's *t*‐test was used to assess differences between the two groups. One‐way analysis of variance and Tukey's post‐hoc test were used to assess differences among three or more groups. A *p*‐value of <.05 was considered statistically significant.

## 3 RESULTS

### 3.1 The effect of quercetin on spatial learning and memory in HIBD mice

**FIGURE 1:**
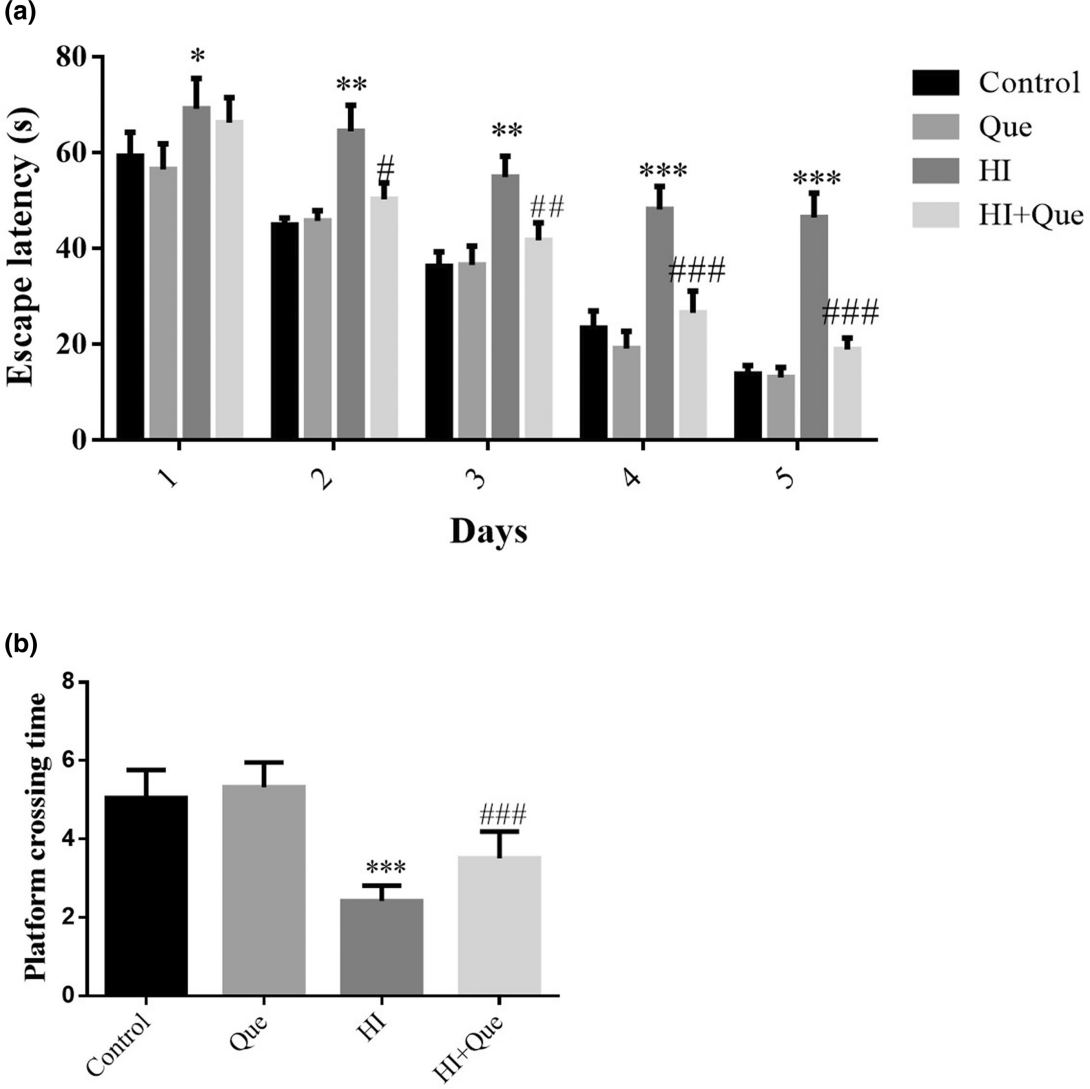
The Escape latency and Platform crossing time of difference groups. Control: The neonatal rats were treated with normal; Que: The neonatal rats were Que (30 mg/kg); HI: The neonatal rats taken HIBD model; HI + Que: The neonatal rats with HIBD were treated with Que (30 mg/kg). (a) Escape latency of different groups. (b) Platform crossing time. *p < .05; **p < .01, ***p < .001, compared with Control group; ^#^ *p* < .05, ^##^ *p* < .01; ^###^ *p* < .001, compared with HI group.

The Morris water maze task was performed to measure the average escape latency and platform crossing times of the mice in each group. On days 1, 2, 3, 4, and 5, the average escape latency of the Que group was significantly higher than that of the control group; however, compared to the HI group, the escape latency of the HI + Que group was significantly lower (*p* < .05) on days 2, 3, 4 and 5 (Figure 1a). According to the spatial probe trial, the platform crossing times in the HI group were significantly lower (*p* < .001) relative to the control group, whereas those of the HI + Que group were significantly greater (*p* < .001) than those of the HI group (Figure 1b). These results suggest that Que improved long‐term learning and memory abilities in HIBD mice.

### 3.2 TTC staining to assess brain infarction

**FIGURE 2:**
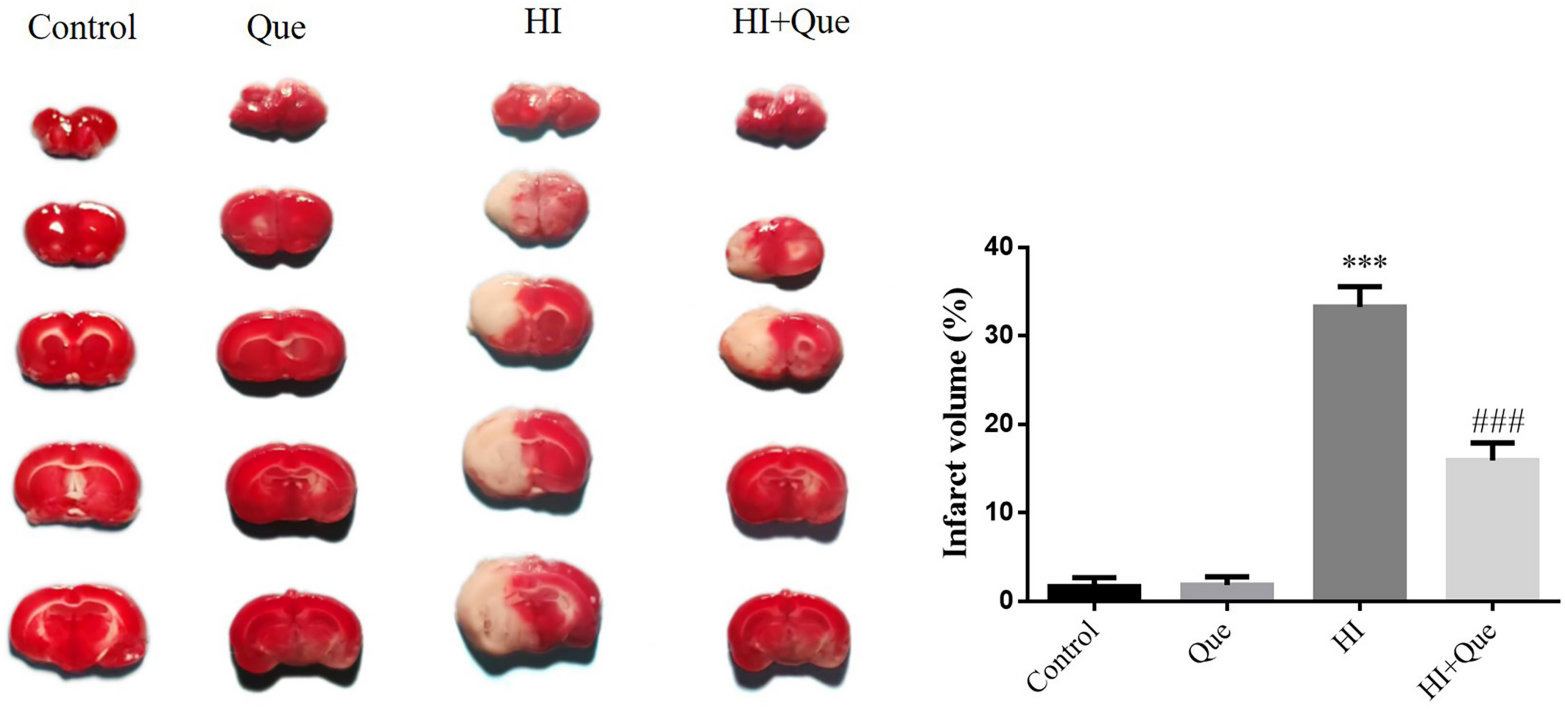
Infarct volume of different groups by TTC staining (%). Control: The neonatal rats were treated with normal; Que: The neonatal rats were Que (30 mg/kg); HI: The neonatal rats taken HIBD model; HI + Que: The neonatal rats with HIBD were treated with Que (30 mg/kg). ***p < .001, compared with Control group; ^###^ *p* < .001, compared with HI group.

2,3,5‐triphenyltetrazolium chloride staining was performed to assess brain infarction in each group of mice. White unstained areas indicated infarction in the brain tissues of mice after hypoxia–ischemia. After the Que intervention, the infarct area in the HI + Que group was significantly lower (*p* < .001) compared to the HI group (Figure 2).

### 3.3 Effect of quercetin on the morphology of HIBD neonatal rat brains

**FIGURE 3:**
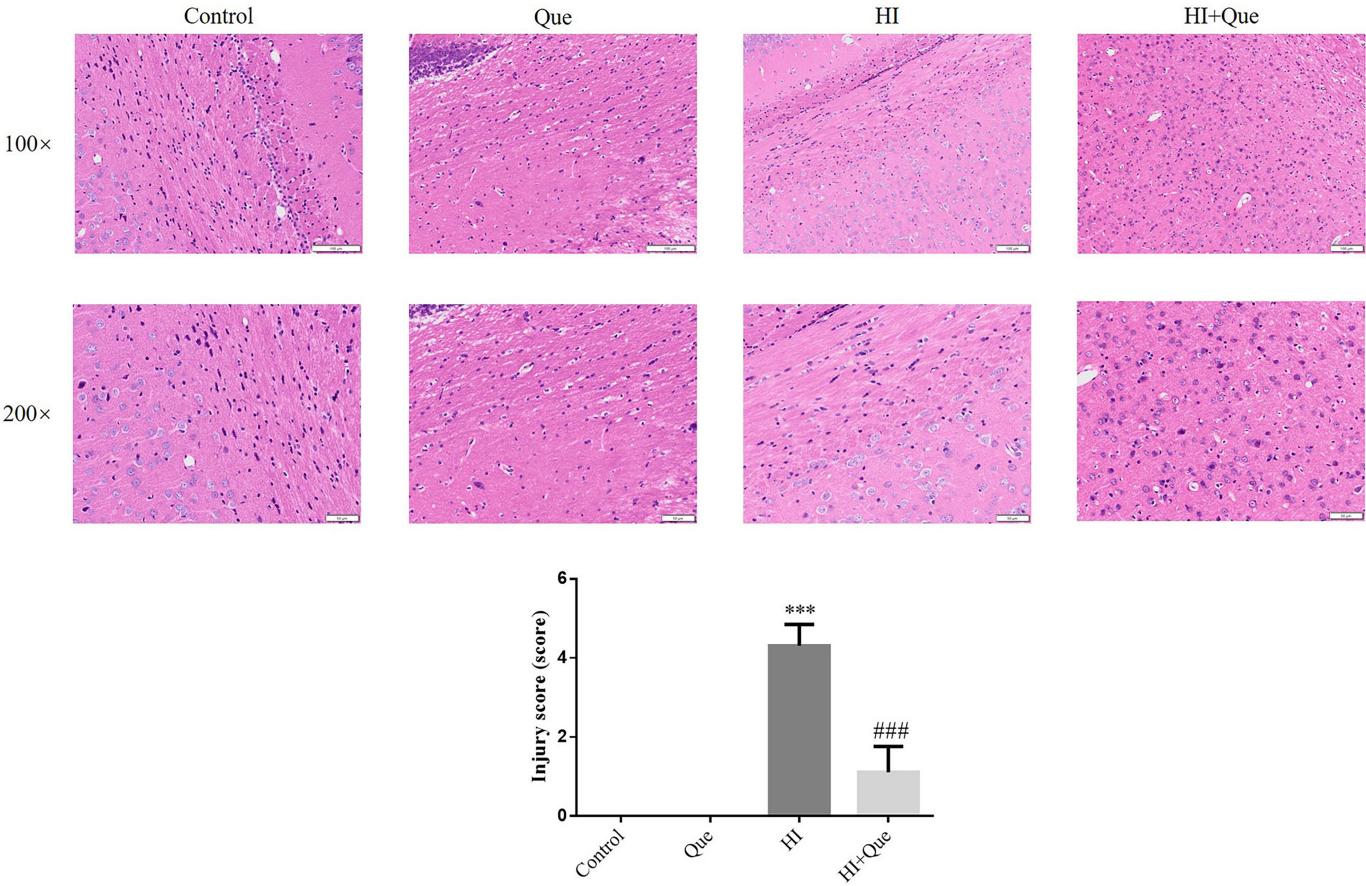
Pathological change in brain tissue of neonatal rats by HE staining (100×, 200×). Control: The neonatal rats were treated with normal; Que: The neonatal rats were Que (30 mg/kg); HI: The neonatal rats taken HIBD model; HI + Que: The neonatal rats with HIBD were treated with Que (30 mg/kg). ***p < .001, compared with Control group; ^###^ *p* < .001, compared with HI group.

H&E staining was used to evaluate the morphological changes in brain tissues (Figure 3). The cerebral cortex in the control and Que groups was thick and full, with orderly neuron arrangements; no obvious structural abnormalities were observed. However, in the HI group, the cortical layers exhibited disordered cell arrangement, neural degeneration and necrosis, widened pericellular spaces, and disrupted structural layers. The cortical tissue damage in the HI + Que group was less severe compared to the HI group. Compared with the control, the injury score significantly increased (*p* < .001) in the HI group (Figure 3). Notably, the injury score of the HI + Que group was significantly lower (*p* < .001) than that of the HI group (Figure 3).

### 3.4 Effect of quercetin on the number of mitochondria in neonatal rat brain tissues

**FIGURE 4:**
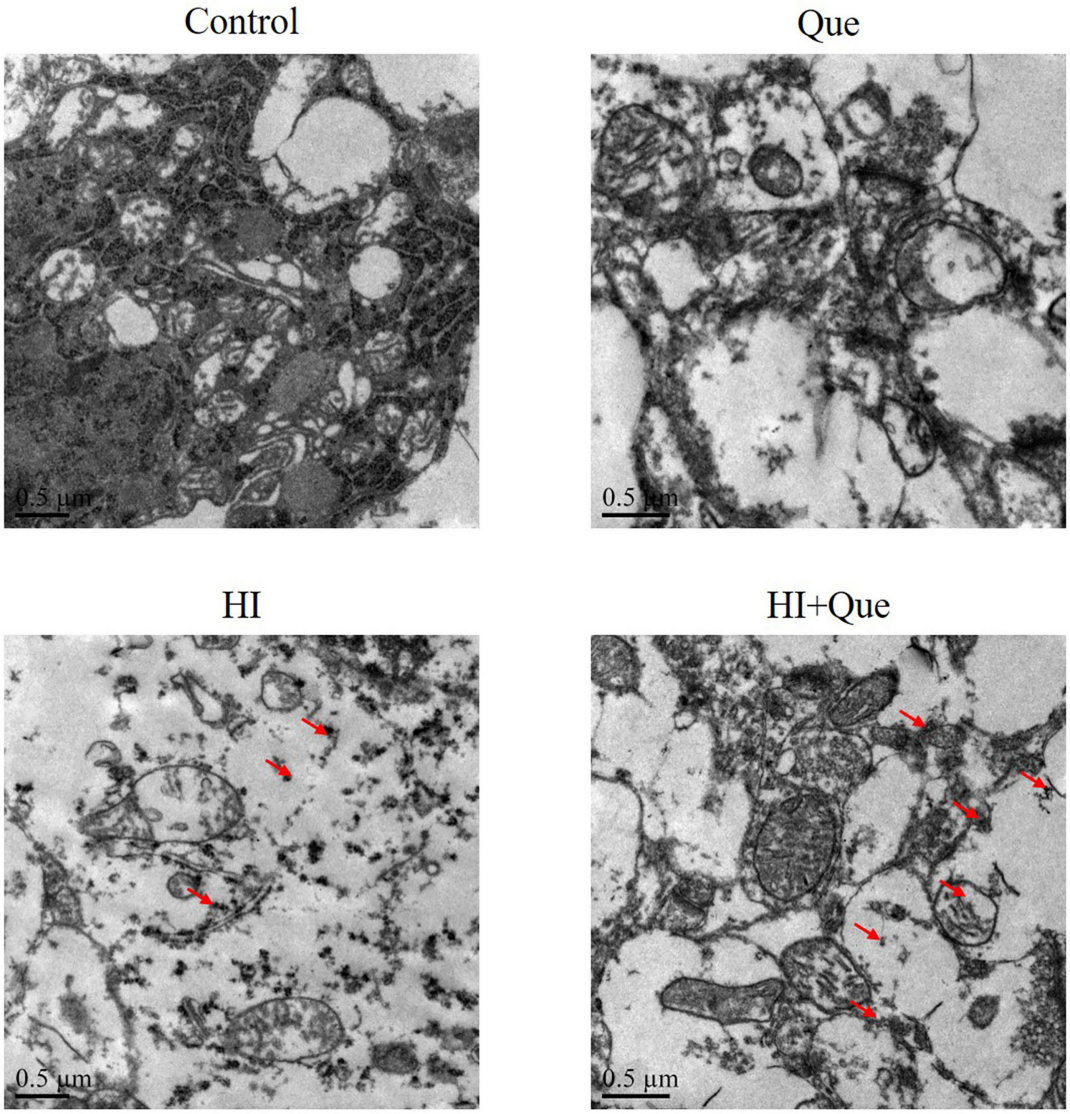
The ultra‐microstructure of neonatal rats' brain tissues by TEM (20000×). Control: The neonatal ats were treated with normal; Que: The neonatal rats were Que (30 mg/kg); HI: The neonatal rat taken HIBD model; HI + Que: The neonatal rats with HIBD were treated with Que (30 mg/kg). Red arrow: Autophagosome.

According to TME results (Figure 4), few autophagosomes were present in the mitochondria of brain tissues in the control and Que groups. In contrast, the number of autophagosomes in the mitochondria of brain tissues in the HI group increased significantly. After Que intervention, the number of autophagosomes further increased significantly compared to the HI group (Figure 4).

### 3.5 Effect of quercetin on apoptosis in neonatal rat brain tissues with HIBD

**FIGURE 5:**
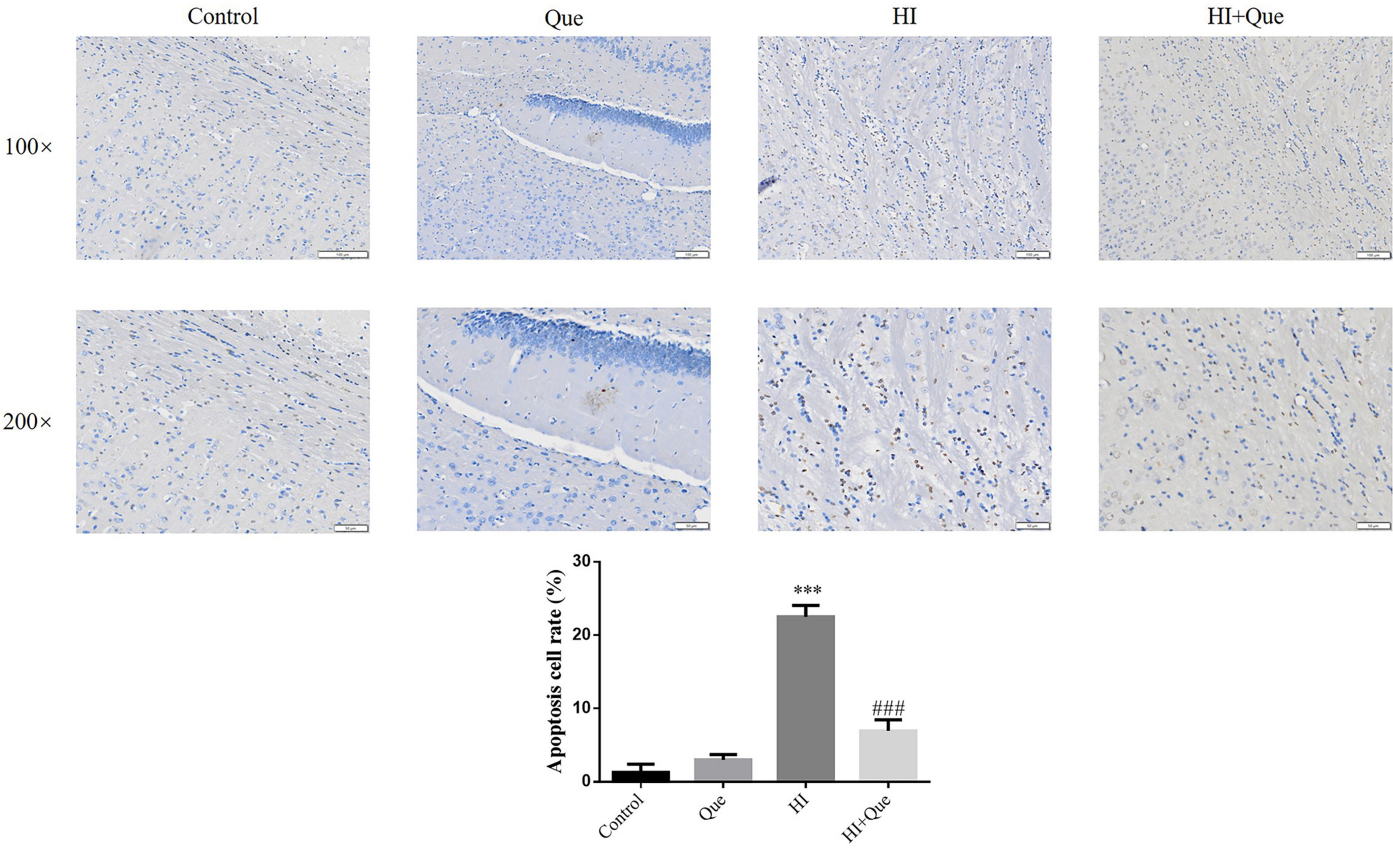
Apoptosis cell rats in brain tissue of neonatal rats by TUNEL (100×, 200×). Control: The neonatal rats were treated with normal; Que: The neonatal rats were Que (30 mg/kg); HI: The neonatal rats taken HIBD model; HI + Que: The neonatal rats with HIBD were treated with Que (30 mg/kg). ***p < .001, compared with Control group; ^###^ *p* < .001, compared with HI group.

TUNEL staining was performed to quantify apoptotic cells (Figure 5). No apoptotic cells were observed in the brain tissues of the control and Que groups. However, the number of apoptotic cells in the HI group significantly increased (*p* < .001; Figure 5). The number of apoptotic cells in the HI + Que group was significantly lower (*p* < .001) than that of the HI group (Figure 5).

### 3.6 Effect of quercetin on gene expression in neonatal rat brain tissues with HIBD

**FIGURE 6:**
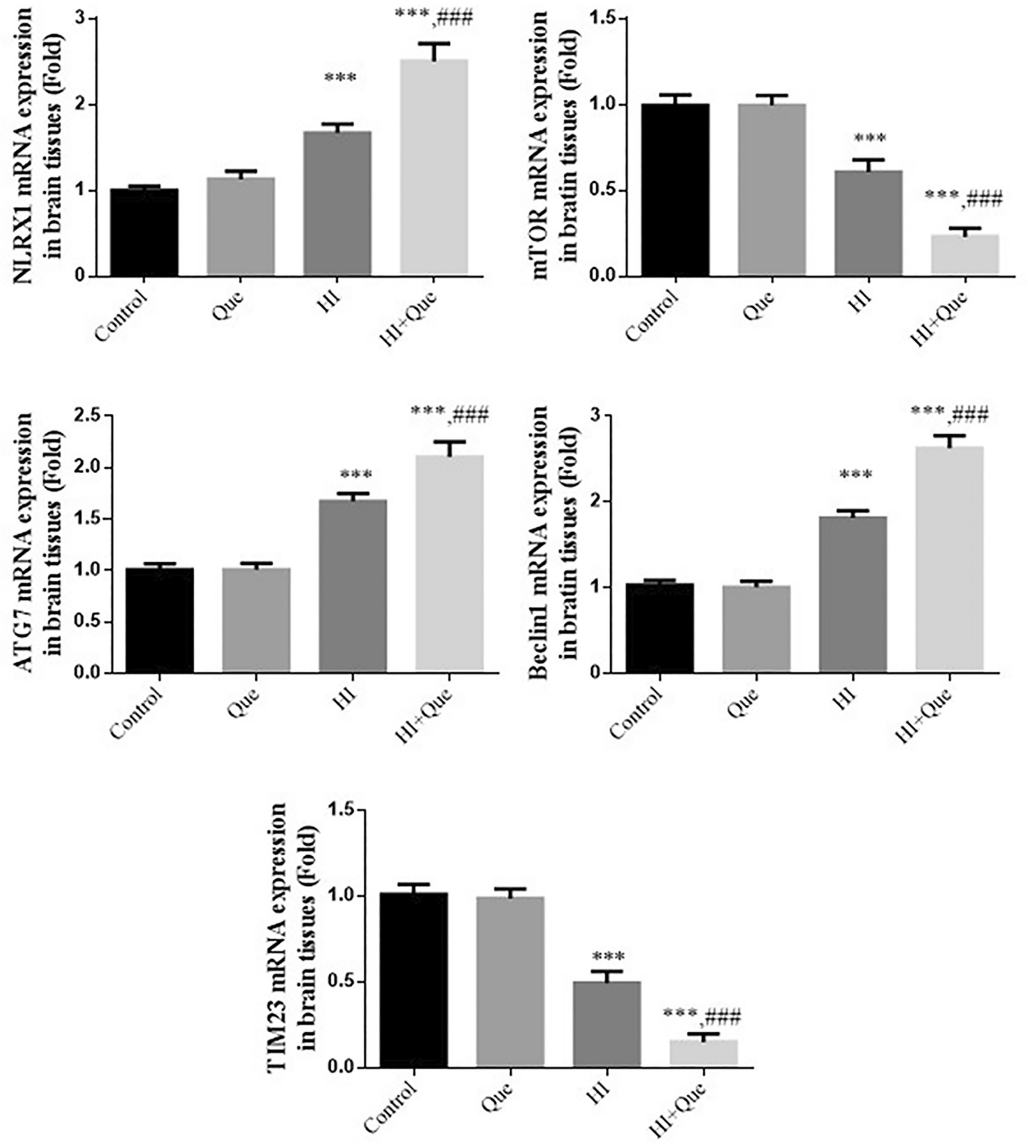
Relative mRNA expression in brain tissues by RT‐qPCR assay. Control: The neonatal rats were treated with normal; Que: The neonatal rats were Que (30 mg/kg); HI: The neonatal rats taken HIBD model; HI + Que: The neonatal rats with HIBD were treated with Que (30 mg/kg). ***p < .001, compared with Control group; ^###^ *p* < .001, compared with HI group.

According to RT‐qPCR, the expression levels of *NLRX1*, *ATG7*, and *Beclin1* were significantly upregulated relative to the control group, whereas *mTOR* and *TIM23* were significantly downregulated (*p* < .001) in both the HI and HI + Que groups (Figure 6). Gene expression levels of *NLRX1*, *ATG*, *Beclin1*, *mTOR*, and *TIM23* significantly differed (*p* < .001) between the HI and HI + Que groups (Figure 6).

### 3.7 Effect of quercetin on protein expression in neonatal rat brain tissues

**FIGURE 7:**
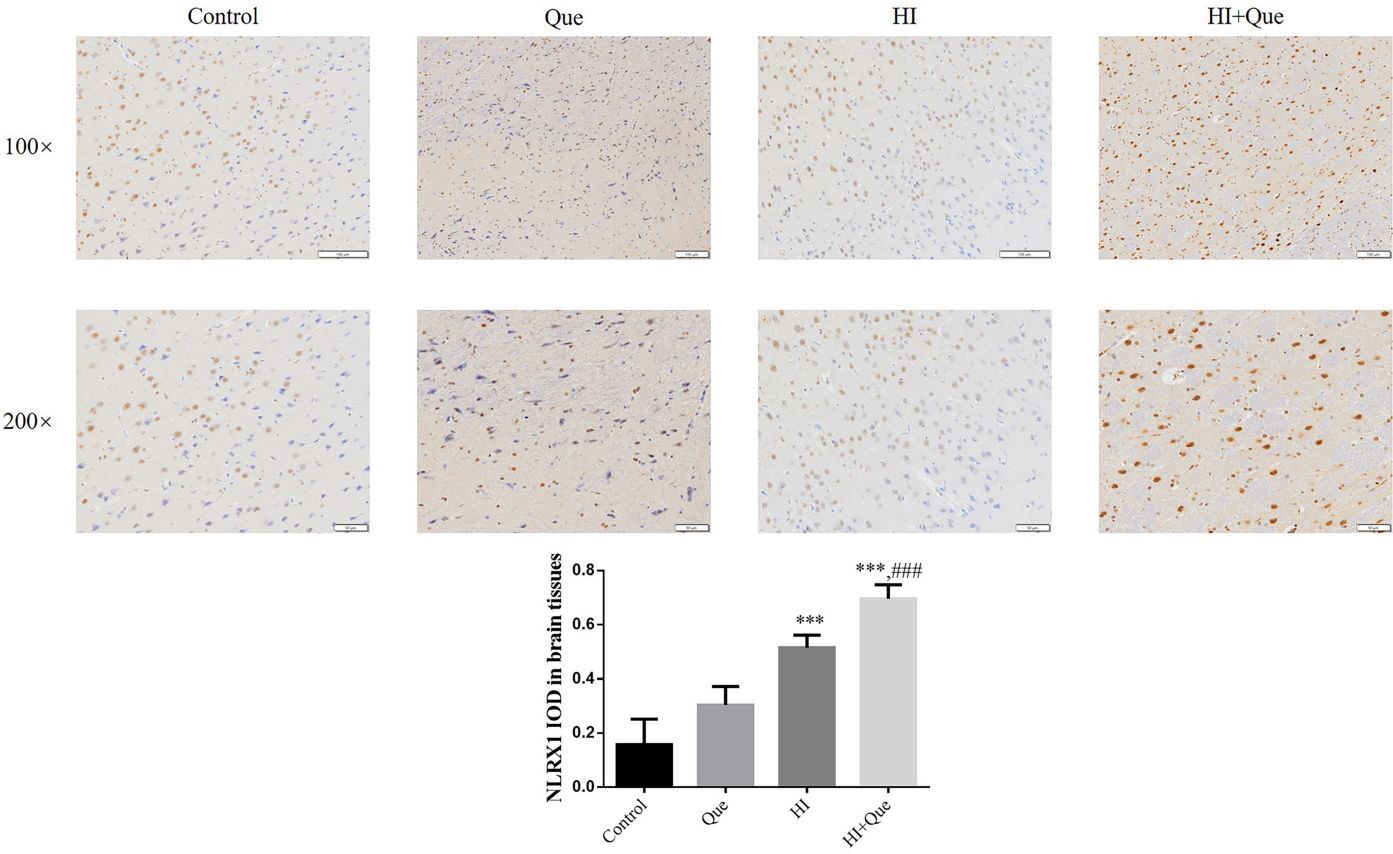
NLRX1 protein expression in brain tissues by IHC assay (100×, 200×). Control: The neonatal rats were treated with normal; Que: The neonatal rats were Que (30 mg/kg); HI: The neonatal rats taken HIBD model; HI + Que: The neonatal rats with HIBD were treated with Que (30 mg/kg). ***p < .001, compared with Control group; ^###^ *p* < .001, compared with HI group.

**FIGURE 8:**
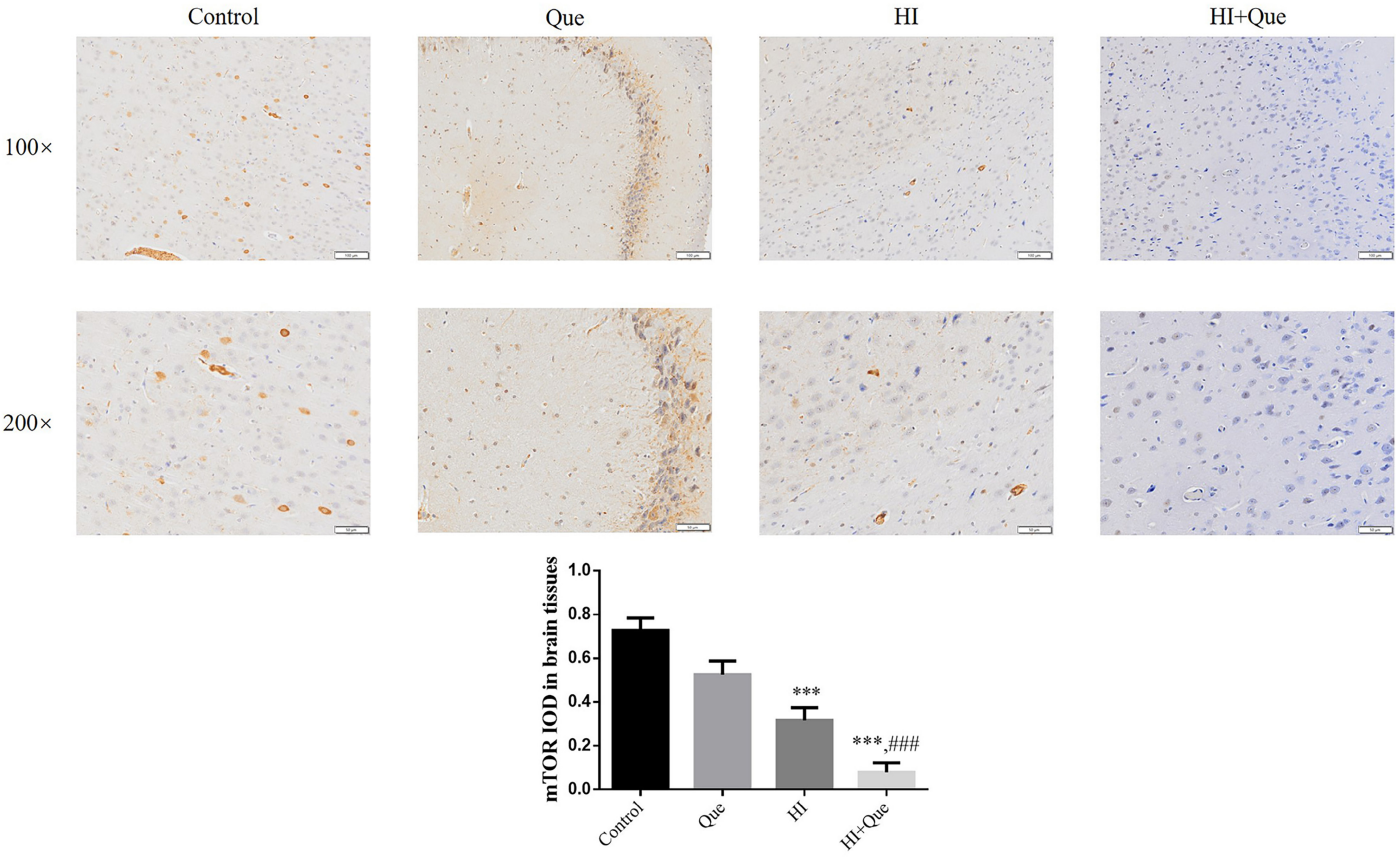
mTOR protein expression in brain tissues by IHC assay (100×, 200×). Control: The neonatal rats were treated with normal; Que: The neonatal rats were Que (30 mg/kg); HI: The neonatal rats taken HIBD model; HI + Que: The neonatal rats with HIBD were treated with Que (30 mg/kg). ***p < .001, compared with Control group; ^###^ *p* < .001, compared with HI group.

**FIGURE 9:**
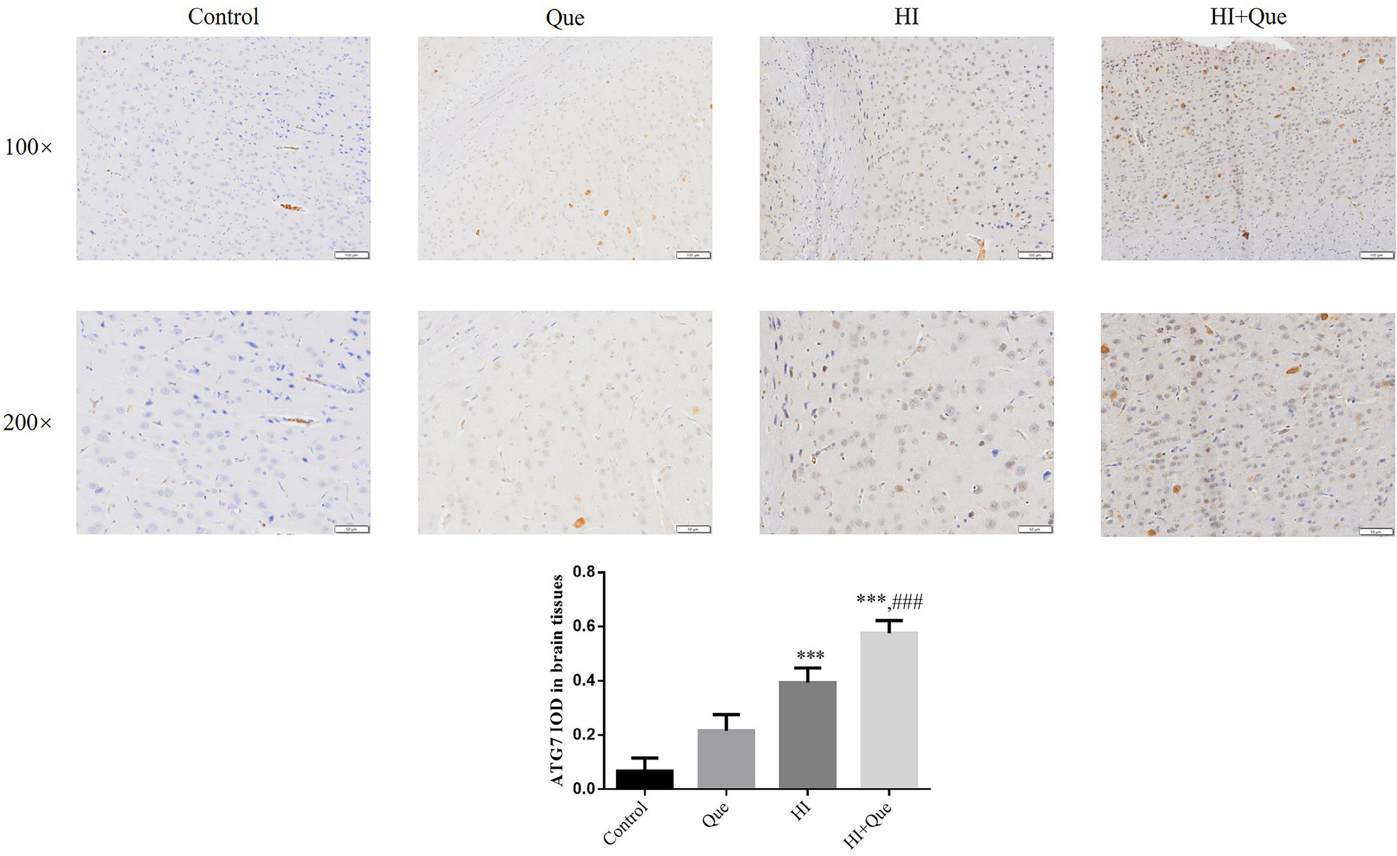
ATG7 protein expression in brain tissues by IHC assay (100×, 200×). Control: The neonatal rats were treated with normal; Que: The neonatal rats were Que (30 mg/kg); HI: The neonatal rats taken HIBD model; HI + Que: The neonatal rats with HIBD were treated with Que (30 mg/kg). ***p < .001, compared with Control group; ^###^ *p* < .001, compared with HI group.

**FIGURE 10:**
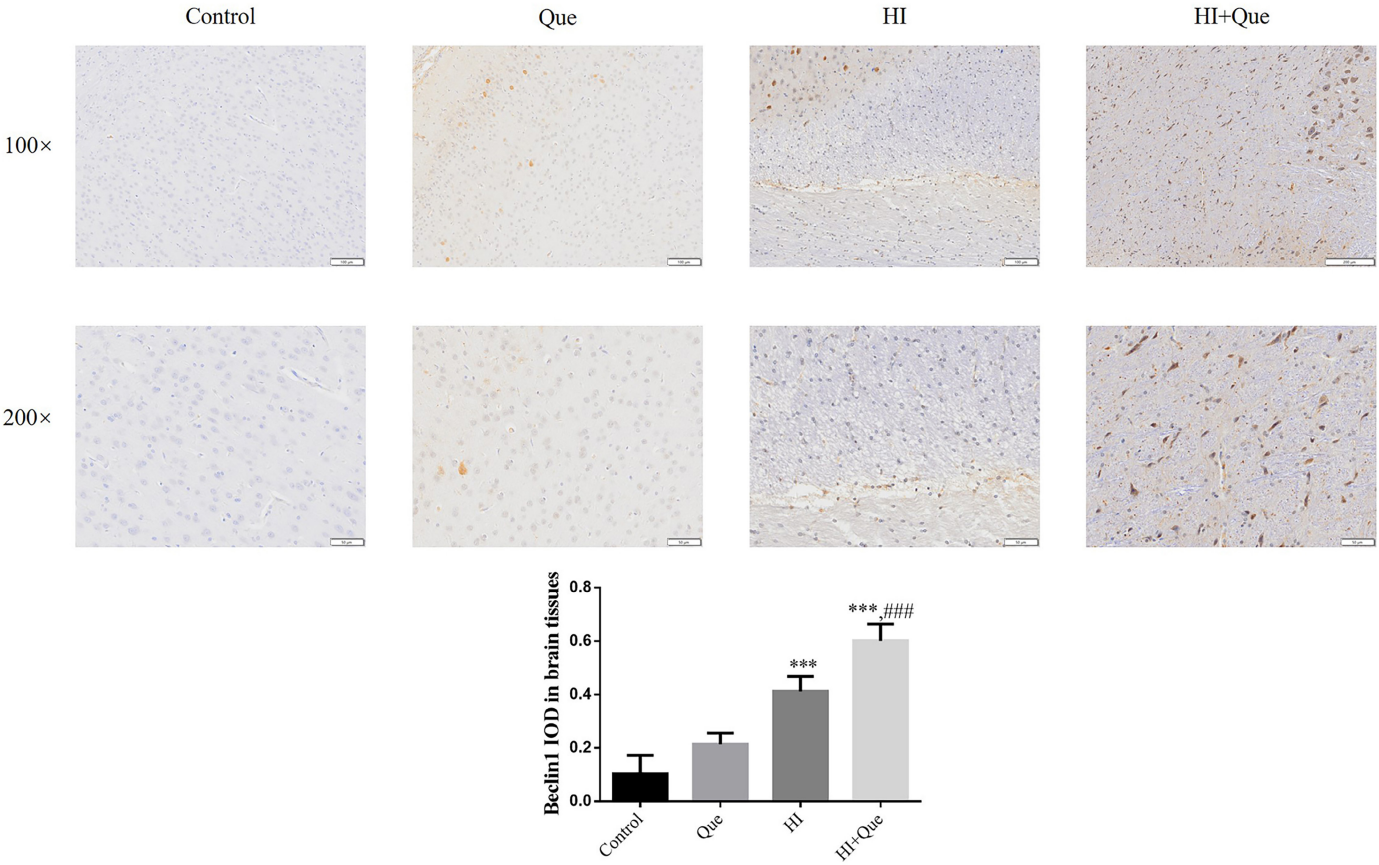
Beclin1 protein expression in brain tissues by IHC assay (100×, 200×). Control: The neonatal rats were treated with normal; Que: The neonatal rats were Que (30 mg/kg); HI: The neonatal rats taken HIBD model; HI + Que: The neonatal rats with HIBD were treated with Que (30 mg/kg). ***p < .001, compared with Control group; ^###^ *p* < .001, compared with HI group.

**FIGURE 11:**
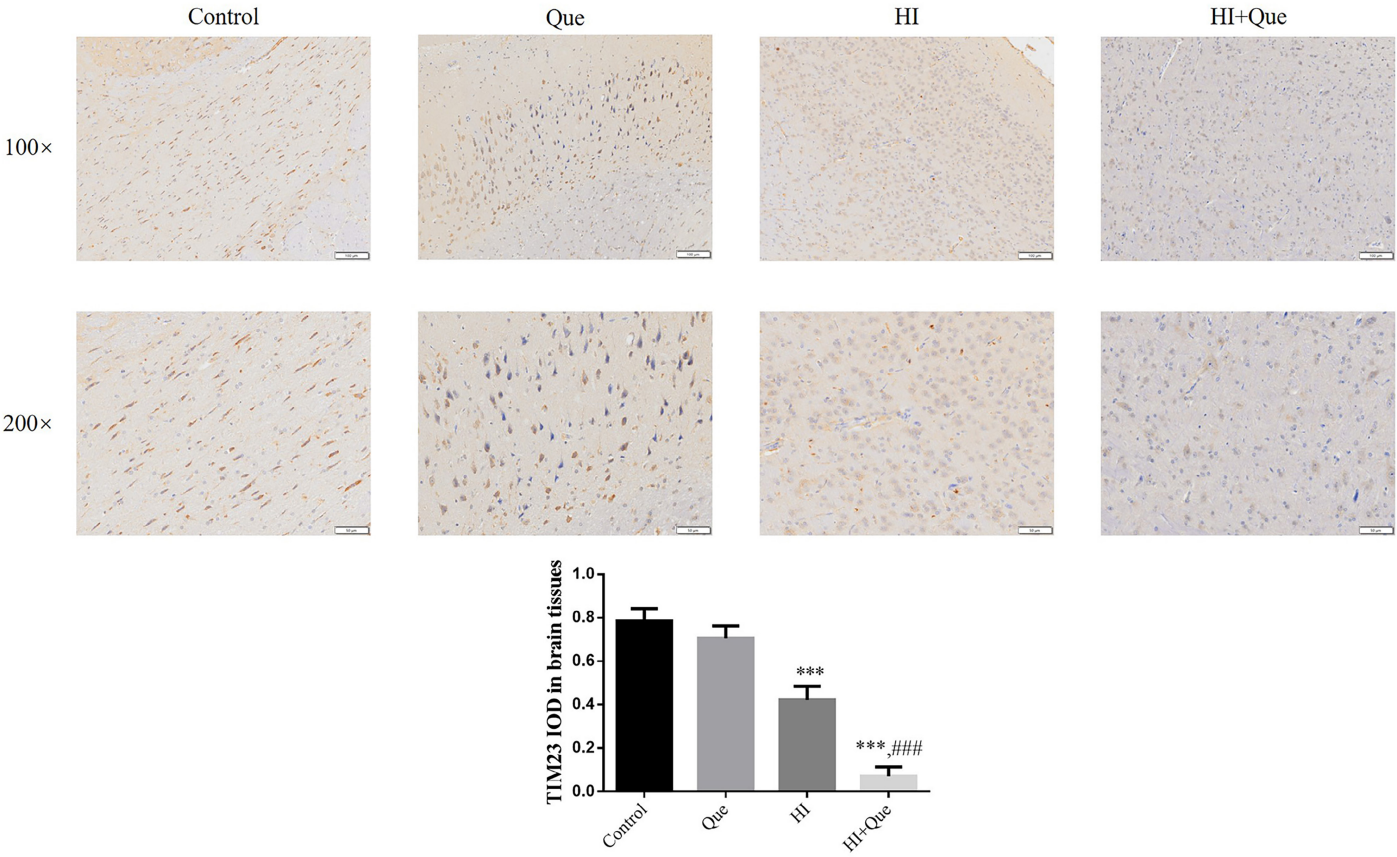
TIM23 protein expression in brain tissues by IHC assay (100×, 200×). Control: The neonatal rats were treated with normal; Que: The neonatal rats were Que (30 mg/kg); HI: The neonatal rat taken HIBD model; HI + Que: The neonatal rats with HIBD were treated with Que (30 mg/kg). ***p < .001, compared with Control group; ^###^ *p* < .001, compared with HI group.

IHC results revealed significantly increased expression levels of NLRX1, ATG7, and Beclin1 proteins, but mTOR and TIM23 protein expression levels were significantly lower (*p* < .001) in both the HI and HI + Que groups (Figures 7, 8, 9, 10, 11). Significant differences (*p* < .001) in the expression levels of NLRX1, ATG, Beclin1, mTOR, and TIM23 proteins were observed between the HI and HI + Que groups (Figures 7, 8, 9, 10, 11).

### 3.8 Effect of quercetin on LC3B protein expression in neonatal rat brain tissues with HIBD based on immunofluorescence detection

**FIGURE 12:**
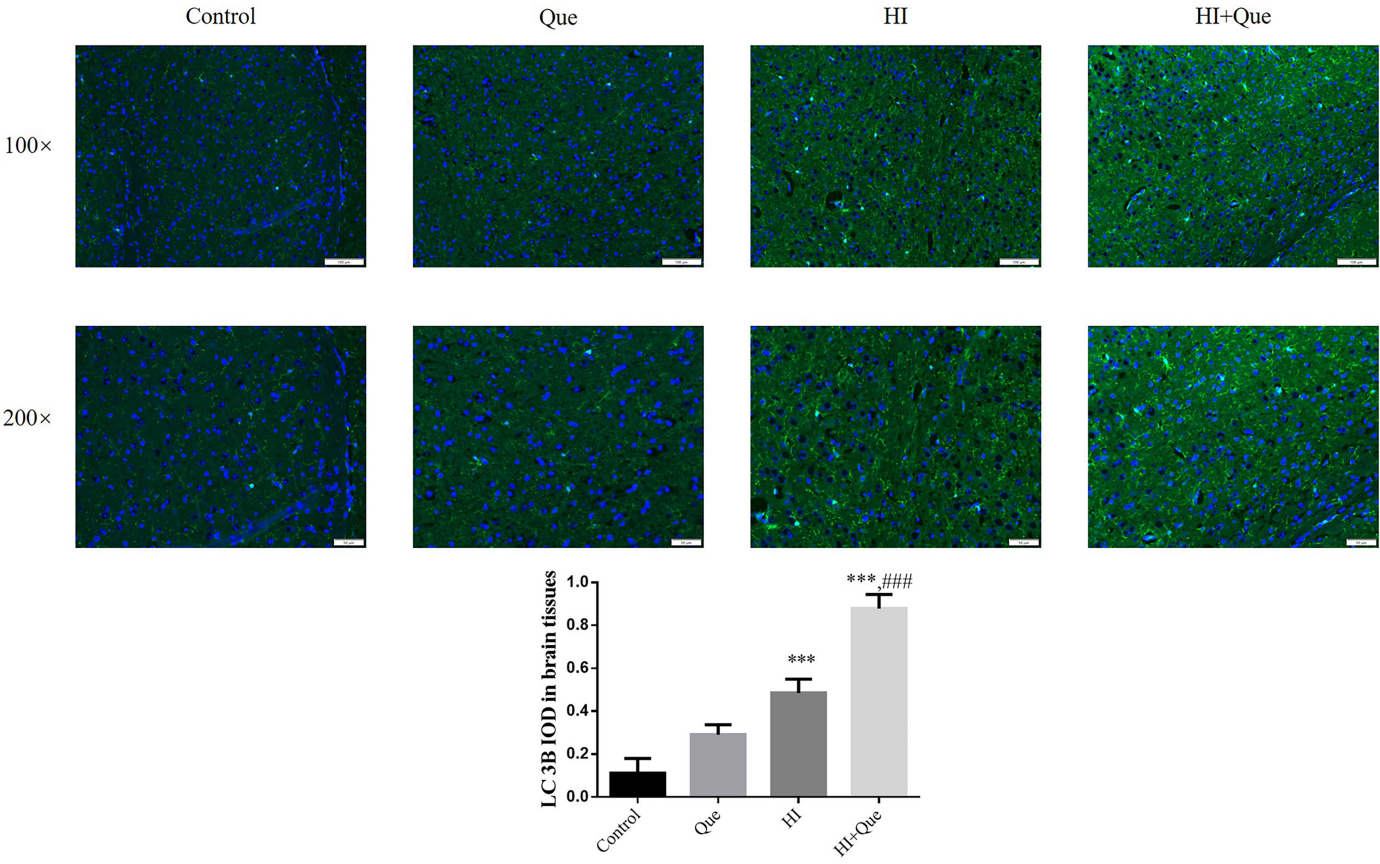
LC 3B protein expression in brain tissues by IF assay (100×, 200×). Control: The neonatal rats were treated with normal; Que: The neonatal rats were Que (30 mg/kg); HI: The neonatal rats taken HIBD model; HI + Que: The neonatal rats with HIBD were treated with Que (30 mg/kg). ***p < .001, compared with Control group; ^###^ *p* < .001, compared with HI group.

Immunofluorescence staining revealed significantly greater (*p* < .001) expression of LC3B protein in both the HI and HI + Que groups relative to the control (Figure 12). Significant differences (*p* < .001) in LC3B protein expression levels were observed between the HI and HI + Que groups (Figure 12).

### 3.9 Effect of quercetin on relative protein expression in neonatal rat brain tissues with HIBD based on western blot

**FIGURE 13:**
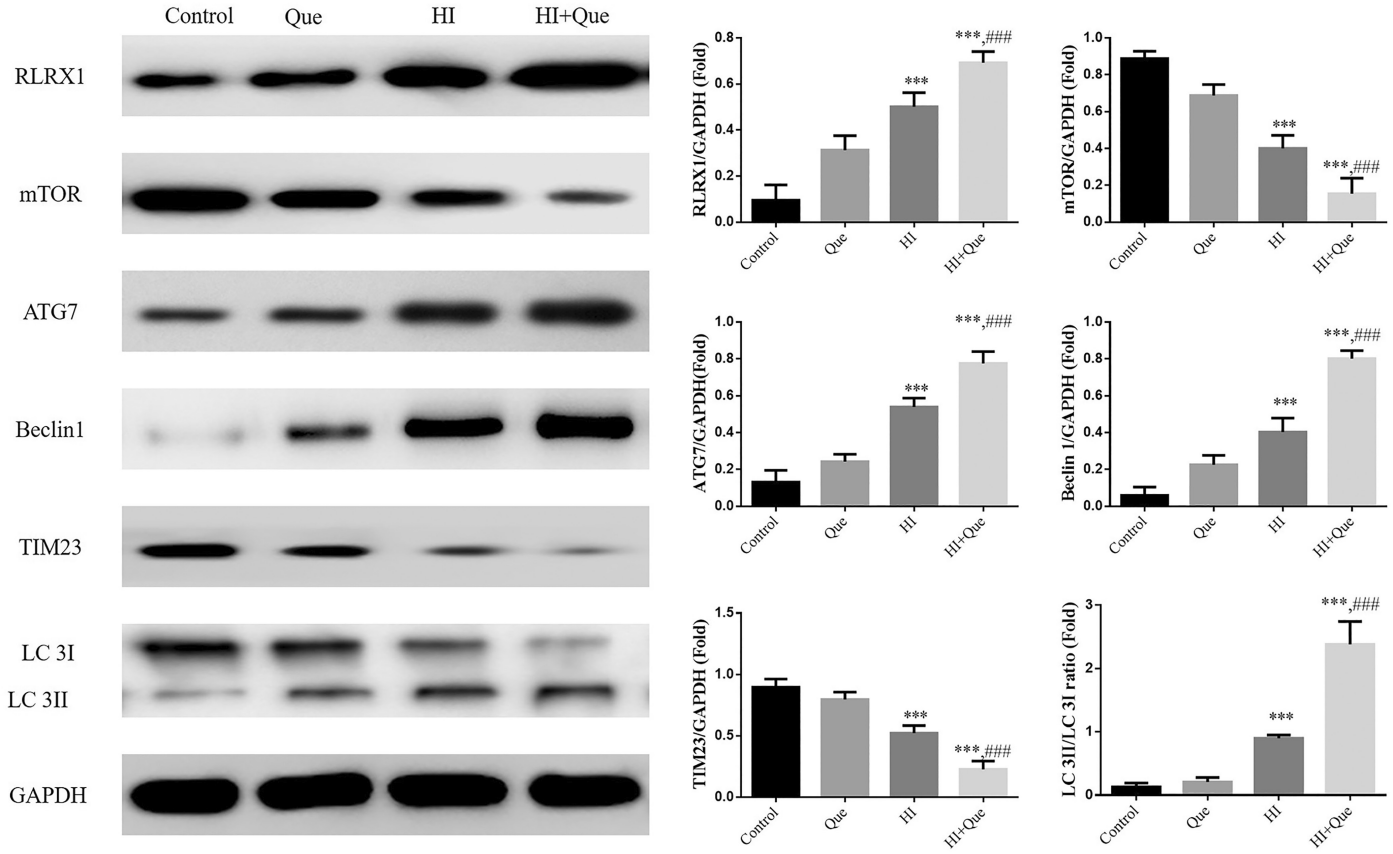
Relative protein expression in brain tissues by WB assay. Control: The neonatal rats were treated with normal; Que: The neonatal rats were Que (30 mg/kg); HI: The neonatal rat taken HIBD model; HI + Que: The neonatal rats with HIBD were treated with Que (30 mg/kg). ***p < .001, compared with Control group; ^###^ *p* < .001, compared with HI group.

According to WB analysis, RLRX1, ATG7, and Beclin1 proteins, as well as the LC 3II/LC 3I ratio, were significantly upregulated (*p* < .001) in the HI group, whereas TIM23 protein expression was significantly downregulated (*p* < .001; Figure 13) compared to the control group. Compared with the HI group, RLRX1, ATG7, and Beclin1 proteins, as well as the LC 3II/LC 3I ratio, were significantly upregulated (*p* < .001), and TIM23 protein expression significantly downregulated (*p* < .001) in the HI + Que group (Figure 13).

## 4 DISCUSSION

Perinatal hypoxic‐ischemic events are a major cause of neonatal brain injury. Although hypoxia–ischemia‐induced damage to peripheral organs is generally reversible, it can permanently injure the central nervous system. Ischemic events may occur during delivery and include umbilical cord prolapse, placental abruption, uterine rupture, dystocia, and fetal infections; perfusion and oxygenation may affect vital organs in fetuses and neonates as well (Yıldız et al., 2017). Prenatal and neonatal care has improved in recent years, yet hypoxic–ischemic brain injury remains a leading cause of neonatal morbidity and mortality worldwide, with an incidence of up to 26 per 1000 live births in developing countries (Kurinczuk et al., 2010). Some infants with HIE die during the neonatal period, whereas survivors may later experience intellectual disability, epilepsy, cerebral palsy, or developmental delay (Douglas‐Escobar & Weiss, 2015; Schreglmann et al., 2020). Currently, therapeutic hypothermia is the only intervention supported by evidence‐based medicine; however, its success rate is limited. Therefore, safe and effective treatment strategies are needed to target the pathogenesis of hypoxic–ischemic brain injury.

Cells and tissues undergo complex changes during the development of neonatal HIBD. Initially, the pathological changes are caused by an insufficient supply of oxygen to cells and tissues, leading to primary energy failure. Subsequently, high‐energy phosphate levels briefly recover, marking the arrival of the second stage, during which excitotoxicity, massive calcium influx, oxidative and nitrosative stress, and inflammation occur. These lead to cell necrosis or apoptosis. The injury ultimately progresses to the third stage, lasting for several weeks or even months (Pregnolato et al., 2019; Torres‐Cuevas et al., 2019). The crux of hypoxia–ischemia lies in energy deficiency and, therefore, in mitochondria, organelles that provide energy to cells and orchestrate the nervous system. Mitochondria respond to hypoxia or ischemia by swelling or mitochondrial fusion (Han et al., 2018). Research on ischemia–reperfusion suggests that ischemia–reperfusion interferes with mitochondrial dynamics and mitophagy, subsequently leading to mitochondrial dysfunction and exacerbating organ injury (Anzell et al., 2018). Indeed, mitochondrial dysfunction has been implicated in various neurological diseases. For example, ROS may contribute to hypoxia‐ or ischemia‐induced neuronal damage (Gao et al., 2017). However, most ROS are produced by damaged mitochondria (Huang et al., 2011). Neurons are highly sensitive to ATP deficiency, and mitochondria ensure that neurons survive under ischemic conditions (Borutaite et al., 2013). Previous studies have shown that oxidative stress due to mitochondrial dysfunction leads to cell death following neonatal HIBD (Niatsetskaya et al., 2012). Damaged and dysfunctional mitochondria can be continuously degraded through mitophagy to reduce oxidative load and be replaced by newly formed mitochondria through biogenesis (Gottlieb & Carreira, 2010). Thus, mitophagy regulates neuronal dynamics. In this study, we explored mitophagy in a neonatal rat HIBD model.

Whether the role of autophagy is beneficial or detrimental during developmental HIBD remains unknown. One study simulated HIBD by injecting newborn rats with glutamate analogs to cause excitotoxic damage and concluded that increased autophagy is harmful (Descloux et al., 2018). Some studies indicate that inhibiting autophagy promotes neuroprotection in the HIE rat model (Zhang et al., 2021; Zhu et al., 2022), but others revealed neuroprotective effects following the activation of autophagy (Ye et al., 2018; Zheng et al., 2018). It has been proposed that a moderate increase in autophagy protects against perinatal HIBD (Kim et al., 2021). In recent years, studies on brain ischemia–reperfusion have discovered that autophagy plays different roles in brain ischemia and subsequent reperfusion. During reperfusion, the protective role of autophagy may be attributed to the clearance of damaged mitochondria associated with mitophagy and the inhibition of apoptosis (Sun et al., 2018; Zhang et al., 2013; Zhang & Yu, 2018). Thus, mitophagy may serve as an endogenous protective mechanism for brain ischemia–reperfusion injury. However, mitophagy is associated with exacerbated brain injury in other models (Lan et al., 2018). Mitophagy can exert neuroprotective effects (Cai et al., 2019), and research on HIBD in newborn rats has found that the induction of mitophagy and neuronal cell death is sex dependent. Impaired clearance of damaged mitochondria may render male rats more susceptible to neuronal death and long‐term neurological and behavioral abnormalities after hypoxia–ischemia compared to female rats, suggesting that mitophagy has neuroprotective effects (Demarest, Waite, et al., 2016). Additionally, studies have confirmed that mitophagy is enhanced in HIBD animal models; its inhibition leads to exacerbated brain injury (Nagai‐Singer et al., 2019). In the present study, hypoxia–ischemia led to the upregulation of autophagy‐related proteins Beclin1 and ATG7, as well as the LC3‐II/LC3‐I ratio, whereas the expression of the mitochondrial inner membrane protein TIM23 was downregulated. TEM revealed a significant increase in autophagosomes, indicating the occurrence of mitophagy in HIBD.

Given its antioxidant, anti‐inflammatory, and antiapoptotic properties, Que may alleviate HIBD in neonates (Wong et al., 2020). Some studies found that Que ameliorates disease‐induced damage by regulating autophagy (Cao et al., 2019; Reyes‐Farias & Carrasco‐Pozo, 2019; Wu et al., 2017). However, whether Que ameliorates HIBD by regulating autophagy remains unknown. Here, Que administration in a rat model of HIBD led to decreased mTOR protein expression and increased autophagosome numbers in injured brain tissue, suggesting that Que and the mTOR pathway promote mitophagy after HIBD. In addition, TUNEL staining showed that Que intervention alleviated apoptosis caused by hypoxia–ischemia, further suggesting its neuroprotective effects. Nevertheless, the specific mechanisms by which Que regulates mitophagy and inhibits apoptosis remain unclear.

NLRX1, as the only NLR family member localized to the mitochondria, plays a unique role in the nervous system (Theus et al., 2017). As a novel mitochondrial receptor, NLRX1 induces mitophagy of *Listeria monocytogenes* (Zhang et al., 2019). Research on intestinal ischemia–reperfusion injury has discovered that NLRX1 regulates mitophagy and inhibits apoptosis (Li et al., 2021). We measured significantly higher protein and mRNA levels of NLRX1 in the brain tissue of the HI group. Considering the regulatory role of NLRX1 in mitophagy and apoptosis, differential expression of NLRX1 may be related to HIBD‐induced mitophagy. Meanwhile, Que intervention further increased the expression of NLRX1 in the brain tissue of HIBD neonatal rats in the present study, suggesting that it regulates the expression of NLRX1 in the brains of hypoxic–ischemic rats. This encourages further studies on how NLRX1 regulates mitophagy and apoptosis in HIBD as well as on Que's neuroprotective mechanisms.

In conclusion, this study showed that Que may alleviate HIBD‐induced neurodegeneration by regulating autophagy and the expression of NLRX1.

## AUTHOR CONTRIBUTIONS

**Yan‐hong Xu:** Investigation (equal); methodology (equal). **Jin‐bo Xu:** Software (equal); supervision (equal). **Lu‐lu Chen:** Conceptualization (equal); software (equal). **Wei Su:** Methodology (equal); supervision (equal). **Qing Zhu:** Funding acquisition (equal); visualization (equal). **Guang‐lei Tong:** Funding acquisition (equal); supervision (equal); validation (equal).

## CONFLICT OF INTEREST STATEMENT

There is no conflict of interest Statement in this research.

## ETHICS STATEMENT

This study was approved by Ethics Committee of Anhui Provincial Children's Hospital (No. APC2021060723).

## PATIENT CONSENT FOR PUBLICATION

Not applicable.

## Data Availability

All data belong to the corresponding author. If you need data, please contact the corresponding author (E‐mail: tongguanglei0323@163.com).
